# Independent genetic factors control floret number and spikelet number in *Triticum turgidum* ssp.

**DOI:** 10.3389/fpls.2024.1390401

**Published:** 2024-08-26

**Authors:** Kiros A. Y., Mica E., Battaglia R., Mazzucotelli E., Dell’Acqua M., Cattivelli L., Desiderio F.

**Affiliations:** ^1^ Center of Plant Sciences, Scuola Superiore Sant’Anna, Pisa, Italy; ^2^ Council for Agricultural Research and Economics (CREA) – Research Centre for Genomics and Bioinformatics, Fiorenzuola d’Arda, Italy; ^3^ Dipartimento per lo Sviluppo Sostenibile e la Transizione Ecologica, Università del Piemonte Orientale, Vercelli, Italy

**Keywords:** durum wheat, *T. dicoccum*, spike architecture, florets, QTLs, yield

## Abstract

Wheat grain yield is a complex trait resulting from a trade-off among many distinct components. During wheat evolution, domestication events and then modern breeding have strongly increased the yield potential of wheat plants, by enhancing spike fertility. To address the genetic bases of spike fertility in terms of spikelet number per spike and floret number per spikelet, a population of 110 recombinant inbred lines (RILS) obtained crossing a *Triticum turgidum* ssp. *durum* cultivar (Latino) and a *T. dicoccum* accession (MG5323) was exploited. Being a modern durum and a semi-domesticated genotype, respectively, the two parents differ for spike architecture and fertility, and thus the corresponding RIL population is the ideal genetic material to dissect genetic bases of yield components. The RIL population was phenotyped in four environments. Using a high-density SNP genetic map and taking advantage of several genome sequencing available for *Triticeae*, a total of 94 QTLs were identified for the eight traits considered; these QTLs were further reduced to 17 groups, based on their genetic and physical co-location. QTLs controlling floret number per spikelet and spikelet number per spike mapped in non-overlapping chromosomal regions, suggesting that independent genetic factors determine these fertility-related traits. The physical intervals of QTL groups were considered for possible co-location with known genes functionally involved in spike fertility traits and with yield-related QTLs previously mapped in tetraploid wheat. The most interesting result concerns a QTL group on chromosome 5B, associated with spikelet number per spike, since it could host genes still uncharacterized for their association to spike fertility. Finally, we identified two different regions where the trade-off between fertility related traits and kernel weight is overcome. Further analyses of these regions could pave the way for a future identification of new genetic loci contributing to fertility traits essential for yield improvement in durum wheat.

## Introduction

Durum wheat (*Triticum turgidum L*. ssp. *durum*) is a tetraploid wheat species used to produce semolina, a raw material for preparing pasta and couscous. Although it accounts for only 5 to 8 percent (Martínez-Moreno et al., 2022) of total wheat world production, durum wheat represents a fundamental food source for countries of the Mediterranean basin and, together with its tetraploid progenitors, a source of genetic diversity for wheat improvement. Durum wheat is grown on approximately 16 million hectares globally and yields about 38 million tons of grains every year (Martínez-Moreno et al., 2020).

Increasing durum wheat yield through breeding programs is crucial to meet the ever-growing demand of the world’s population for the decades to come. Grain yield (GY) is a complex quantitative trait influenced by multiple genes and their interaction with environmental factors (Sakuma and Schnurbusch, 2020). When GY is dissected into more discrete yield components, a higher heritability is observed (Wang et al., 2018; Zhang et al., 2018; Brinton and Uauy, 2019). Potential yield is determined by two main components, namely grain number and grain weight (Slafer et al., 2022), which are in turn affected by additional sub-components, as the number of spikes per plant, the number of grains per spike, and the grain size. Furthermore, the number of grains per spike can be considered a proxy of spike fertility, which in turn depends on the number of spikelets per spike and the number of fertile florets per spikelet. The only drawback of this relationship is floret primordia abortion that may impair a full grain setting (González-Navarro et al., 2015). On the other hand, the negative correlation existing between grain number and grain weight represents the main obstacle that breeders have to face to increase grain yield, by reaching the best trade-off between the two yield components (Acreche and Slafer, 2006; Bustos et al., 2013; García et al., 2013; Quintero et al., 2018).

At maturity, the wheat spike, organized around the rachis, carries a series of alternating spikelets, each differentiating around a rachilla which carries multiple florets. Inflorescence architecture is one of the traits affected by crop domestication and by breeding for grain yield improvement (Gauley and Boden, 2018). The inflorescence is the result of successive changes in meristem identity (Gao et al., 2019). During plant development, the entrance into the reproductive phase from the vegetative stage is marked by the meristem transition from the vegetative to the inflorescence meristem (IM): at this stage the wheat shoot apical meristem (SAM) appears in the typical double ridge conformation. Later the meristem elongates in a spindle shaped structure, forming glume primordia and defining the spikelet meristems (SM). During tillering stage, each spikelet will form multiple floret meristems (FM) that will later differentiate the floral organs. When the terminal apical spikelet is formed, the spike has completed the spikelet initiation phase, and the final number of spikelets per spike is defined (Kirby and Appleyard, 1987). In the stem elongation phase, from the terminal spike stage up to anthesis, the number of total florets and fertile florets in each spikelet is set (Fischer, 1985; Slafer and Rawson, 1994; Miralles and Slafer, 2007; González et al., 2011; González-Navarro et al., 2015). As a consequence, the duration of the tillering stage as well as the rate of spikelet development affect the final number of spikelets. Opposite to the inflorescence meristem, spikelet meristems are undetermined, meaning they remain potentially active in producing new florets, and that extending the duration of the spikelet’s meristem activity is expected to increase the number of florets per spikelet.

Since spike fertility is strictly related to the duration of each plant growing phase, molecular mechanisms responsible of plant phenology have a direct effect on GY. For this reason, master regulators of vernalization requirement and flowering, as the genes *VERNALIZATION1* (*VRN1*), *VRN2* and *VRN3* (more frequently indicated as *Flowering Locus T, FT1*), and *Photoperiod-1 (PPD-1)*, respectively, often co-localize with grain yield loci (Kitagawa et al., 2012; Pérez-Gianmarco et al., 2019). In temperate cereals, *VRN1*, and *VRN2* genes regulate the activity of *FT1* (Shimada et al., 2009; Tanaka et al., 2018; Liu et al., 2019; Shaw et al., 2019), which is required to initiate the transition of the vegetative meristem toward the reproductive stage (Lv et al., 2014). At the molecular level, a cascade of genes is regulated upon *FT1* action leading to spikelets formation and floral organs differentiation. Furthermore, different combinations of *VRN1* and *PPD-1* alleles determine the rate of spikelet and floret formation thus influencing the final kernel number (Cao et al., 2020).

During wheat domestication from wild emmer (*Triticum turgidum* ssp. *dicoccoides*) to cultivated emmer (*Triticum turgidum* ssp. *dicoccum)* and then to durum wheat (*Triticum turgidum* ssp. *durum*), spikes have undergone deep transformations. The most striking were the loss of rachis fragility and therefore of the seed shattering trait, then the loss of glume toughness making free-threshing grains, and finally the increase of seed size and number per spike (Simons et al., 2006; Peng et al., 2011; Sharma et al., 2019). The final number of kernels per spikelet is strictly two in the cultivated emmer, and up to 4 or 5 in durum wheat. Main genes responsible for the domestication syndrome have been identified (brittle rachis*, Br*, then tenacious glume, *Tg*, and *Q*), in some cases along with causative mutations that lead to fixation of favorable alleles for modern agriculture (Nalam et al., 2006; Simons et al., 2006; Dvorak et al., 2012; Faris, 2014; Faris et al., 2014a; Avni et al., 2017; Debernardi et al., 2017). This notwithstanding, since spike fertility is a polygenic trait, associated to Quantitative Trait Loci (QTL), it is far from being completely elucidated at molecular level across domestication events and modern breeding gains within the durum wheat history.

Different genetic approaches including linkage mapping and genome-wide association studies (GWAS) are being applied to dissect the GY and identify its genetic determinants. For this purpose, interesting genetic materials are inter (sub) specific populations obtained by crossing wild/semi domesticated wheat as *T. dicoccum* with modern durum. A few studies have been published using durum × emmer bi-parental populations to map spike architecture traits and, consequently, yield and domestication- related traits (Sharma et al., 2019; Peters Haugrud et al., 2023; Valladares García et al., 2023; Faris et al., 2014c). Nowadays, the efforts to identify yield related associated QTL are supported by the availability of durum wheat genome sequence (Maccaferri et al., 2019) thus facilitating the identification of the causative genes/alleles that can be further demonstrated by the development of specific mutants.With the aim of elucidating the molecular mechanisms influencing specific GY components, we have exploited a population of Recombinant Inbred Lines (RILs) obtained by crossing the durum wheat cultivar Latino and the emmer accession MG5323, which strongly differ for spike architecture. Our analysis led to the identification of genetic loci influencing the spikelet number per spike and floret number per spikelet.

## Materials and methods

### Plant materials

A Recombinant Inbred Line (RIL) population of 110 lines developed via single-seed descent of F2 plants from a cross between the accession MG5323 of *Triticum turgidum* ssp. *dicoccum* and the durum wheat cultivar (*cv*) “Latino” was used in this study. MG5323 was collected in Armenia and maintained by the National Small Grains Collection (USDA-ARS, Aberdeen, ID, USA). The *cv* Latino (pedigree CAPPELLI/ANHINGA/4/YAKTANA-54//(SEL.14)-NORIN-10/BREVOR/3/ST-64/2*THATCHER) was released by Federconsorzi (Italy) in 1982. The population is maintained at the Research Center for Genomics and Bioinformatics in Fiorenzuola d’Arda, Italy. The high-density genetic map used in this study was previously reported in Desiderio et al. (2014). It includes a total of 10840 SNP markers assembled in 14 linkage groups corresponding to the 14 durum wheat chromosomes.

### Field experiments and phenotypic evaluation

The RIL population and the parental lines were evaluated in Fiorenzuola d’Arda (Italy) during the 2018–2019 (henceforth referred to as F19), 2019–2020 (F20) and 2020-2021 (F21) seasons, and in Pisa (Italy) in 2019–2020 (P20), thus providing phenotypic data from four environments (locations x years). The experimental design based on a randomized complete block design with three replications was implemented at both sites. Fifty seeds for each RIL were sown in two rows 1 m long and 20 cm apart. Standard field management requirements, such as weed control and fertilizing, were applied uniformly to all plants. Heading date (HD) was recorded as number of days from the April 1^st^ to the time when 50% of tillers within a plot had spike emerged from the flag leaf sheet. In the P20 the HD could not be scored due to lockdowns associated with the COVID-19 pandemic. Five fresh spikes and five dry spikes were randomly collected at HD (Waddidgton scale 9.5; Waddington et al., 1983) and at maturity for parental lines and RILs, respectively. Both, fresh and dry spikes were analyzed for total floret number (FRT) and total spikelet number (SPK). FRT was scored specifically on six spikelets from the middle part of the spike, and the four highest floret number were considered and averaged. Only florets with a mature palea were considered as fertile. The total number of spikelets per spike (SPK) was obtained by counting all spikelets, both fertile and unfertile, in five spikes for each genotype and averaging them. Empty spikelets were those not containing any fertile floret. Net floret number (NFRT) and net spikelet number (NSPK) traits were obtained by subtracting the number of unfertile florets and spikelets from the total number of florets and spikelets, respectively. Spike length (SPL), measured from the bottom to the top of the spike excluding awns, and spike weight (SPW) were obtained averaging measurements of five dry spikes. Spike density (SD) was calculated as ratio among the total number of spikelets (SPK) and the spikelet length (SPL). Measuring procedures of the phenotypic traits are described in **Supplementary Table 1**.

### Microscopic observation of spike development

Spike development of the parental lines was observed at the stereomicroscope (ZEISS SteREO Discovery.v8) at different time-points. After seed soaking, seeds were kept at 4°C, in the dark, for 1 week. Seeds were transferred in pots in a growth chamber (20°C, 16/8 light/dark, 80%RH) till the third leaf emergence. Plants were then subjected to a 5 weeks vernalization period (4°C, 10/14 light/dark) followed by 1-week of hardening (15°C, 12/12 light dark). Finally, plants were kept at 20°C (16/8 light/dark, 80%RH) till they reached anthesis. After hardening, we sampled 5-6 plants every two or three days, for both lines, for microscope observation, until the yellow anther stage (Kirby and Appleyard, 1987). We repeated the experiment twice, to have more consistent data.

### Statistical analysis

Descriptive statistics were calculated on the distributions of phenotypic values for single and multi-environment raw data. When not otherwise indicated, the R statistical data processing environment was used (R Core Team, 2022). A Shapiro-Wilk test was performed to assess the normality of the distribution of phenotypes before further analyses. For this purpose, we used R/metan (Olivoto and Lúcio, 2020) and R/dlookr (Ryu, 2022). Pearson’s correlation coefficients (r) between the phenotypic traits, recorded for RIL population, were also computed using the R/rcompanion (Mangiafico, 2023). An analysis of variance (ANOVA) was performed for each environment to estimate the variance components of all scored phenotypic traits using the base R ‘aov’ function.

Multi-environment ANOVA was performed to evaluate the performance of genotypes across tested environments. The model was fitted as follows (Equation 1):


(1) $$\;\;\;y_{ijk}=\mu +\tau_{i}+\;E_{k\;}+\;\tau E_{ik\;}+\;\beta_{jk\;}+\;\epsilon_{ijk}$$

Where *y_ijk_* is the phenotypic response of the *i^th^* genotype in the *j^th^* replication and *k^t^* ^h^ environment, *µ* is the overall population mean, *τ_i_* is the effect of the *i^th^* genotype, *E_k_* is the effect of the *k^th^* environment, *τE_ik_* is the interaction effect between the *i^th^* genotype and the *k^th^* environment, *β_jk_* is the effect of the *j^th^* block within the *k^th^* environment, and *ϵ_ijk_* is the random residual error. For single environments, the model was simplified accordingly.

Best linear unbiased predictions (BLUPs) for each single environment and across environments were calculated using the Restricted/Estimated Maximum Likelihood method implemented in R/ASReml (Butler et al., 2017). All the predictor variables were fitted as random terms of the linear mixed model. The obtained BLUPs were used to perform all subsequent analysis, including QTL mapping.

Multi-environment broad-sense heritability (*H^2^*, Schmidt et al., 2019) was derived from the variance component estimates obtained from the BLUP model as follows (Equation 2):


(2) $$H^{2}=\frac{\sigma_{\tau }^{2}}{\left(\sigma_{\tau }^{2}+\frac{\sigma_{\tau E}^{2}}{n_{E}}+\frac{\sigma_{\epsilon }^{2}}{n_{rep}*n_{E}}\right)}\;$$

where *σ^2^_τ_* stands for genotypic variance, *σ^2^_τE_* is the variance component of genotype by environment interaction and *σ^2^_ϵ_* is the error term. *n_E_* and *n_rep _* represent the number of environments and replications, respectively. The broad sense heritability for a single environment was estimated reducing the formula accordingly.

The coefficient of variance of residuals was computed using the formula (Equation 3):


(3) $$CV_{\epsilon }=\left(\frac{\sqrt{\sigma_{\epsilon }}}{\mu }\;\right)\times 100\;$$

### QTL analysis

QTL mapping analysis was performed using R/qtl (Broman et al., 2003), employing BLUPs values calculated for each phenotypic trait for single and multi-environments, and the procedure described by Desiderio et al. (2019). For each trait, an initial QTL scan was performed using simple interval mapping with a 1-cM step (Lander and Botstein, 1989) and the position of the highest LOD was recorded. A genome-wide significance level of 5% was calculated after 1,000 permutations (Churchill and Doerge, 1994). The position and the effect of the QTL were then estimated using the multiple imputation method (Sen and Churchill, 2001) by executing the “sim.geno” command, followed by the “fitqtl” command. To search additional QTLs, the “addqtl” command was used. If a second QTL was detected, “fitqtl” was used to test a model containing both QTLs and their interaction effect. If both QTL remained significant, the “refineqtl” command was used to re-estimate the QTL positions based on the full model including both QTLs. QTL interactions were analyzed, and significant locus combinations were possibly reported based on F value. The additive effects of QTLs were estimated as half the difference between the phenotypic values of the respective homozygotes. The confidence interval (CI) of each QTL was determined as proposed by Darvasi and Soller (1997). The QTLs were named according to the rule “Q+ trait code.gb + chromosome”, where Q stands for QTL, trait code for the trait acronym reported in **Supplementary Table 1**, and lastly the wheat chromosome on which the corresponding QTL is located. A consecutive number (“.1,.2,.3”) was added to the QTL name when for the same trait two or more QTLs were detected on the same chromosome.

### Analysis of physical regions carrying QTLs

QTLs identified in the present study were projected on the *T. durum* reference genome sequence (*cv* “Svevo”; Maccaferri et al., 2019) to define their physical confidence interval. Peak and flanking markers corresponding to the QTL confidence intervals were located on the reference genome using BLAST search matches of the corresponding SNP flanking sequences.

The physical position retrieved for each QTL was used for different comparative analyses. Firstly, we verified whether known genes, previously published in tetraploid or hexaploid wheats and involved in yield related traits, phenology or domestication, mapped within the QTL regions. Secondly, the physical position of QTLs detected was compared with QTLs previously identified in tetraploid wheat: for this purpose, the tetraploid wheat QTLome (Maccaferri et al., 2019) was updated by a literature survey of publicly available linkage and association mapping studies until December 2023 (Desiderio et al., 2019; Giancaspro et al., 2019; Alemu et al., 2020; Fatiukha et al., 2020; Mangini et al., 2021; Negisho et al., 2022; Gesesse et al., 2023; Jabbour et al., 2023; Peters Haugrud et al., 2023; Sesiz, 2023; Valladares García et al., 2023). Thirdly, the physical region underlying the most significant QTLs was explored to identify those genes, among the annotated High Confidence genes (available at https://figshare.com/s/2629b4b8166217890971) whose predicted function could be related to the analyzed phenotype.

Considering both the genes already known to be involved in yield related traits and the most interesting genes retrieved from Svevo gene annotation, we inspected their genomic sequences to identify polymorphisms between the MG5323 accession and durum wheat, by mapping MG5323 whole genome reads (Valladares García et al., 2023) against the *T. durum* reference genome assembly *cv*. “Svevo” (Maccaferri et al., 2019), which resulted 85% identical to Latino according to SNP data (Mazzucotelli et al., 2020). Gene sequences, including both exons and introns, and their upstream regions (2,000 bp) were considered, subsequently the effect of identified SNPs was predicted through SnpEff (Cingolani et al., 2012).

Additionally, the ExpVIP platform (Ramírez-González et al., 2018) was employed to get information on the transcriptional profile of these genes. To this end, the homologous genes in bread wheat (*cv* Chinese Spring) were retrieved and their expression profiles in different tissues/organs (leaf, grain, root, spike) and different developing stages (seedling, vegetative, reproductive) were reported. Transcript abundances were expressed in log2 (transcript per million, tpm). We then calculated the sum of the expression (log2tpm) of each gene in all experiments regarding leaves (EX-L), roots (EX-R), grains (EX-G) and spikes (EX-S) and all experiments (TOT). We filtered out for genes whose TOT > 2.5; and then we applied the following rules to define “tissue-specificity”: 1) grain specific genes: (EX-L + EX-R + EX-S) = 0, and analogously for spike-specific genes; 2) genes enriched in grains EX-G/(EX-S + EX-L + EX-R) > 100, and analogously for spike-enriched genes; 3) genes repressed into grains: (EX-S + EX-L + EX-R)/EX-G >100, excluding genes where EX-G =0 and EX-S+EX-L+EX-R< 10, and analogously for spike-repressed genes.

## Results

### Phenotypic evaluation

**Figure 1:**
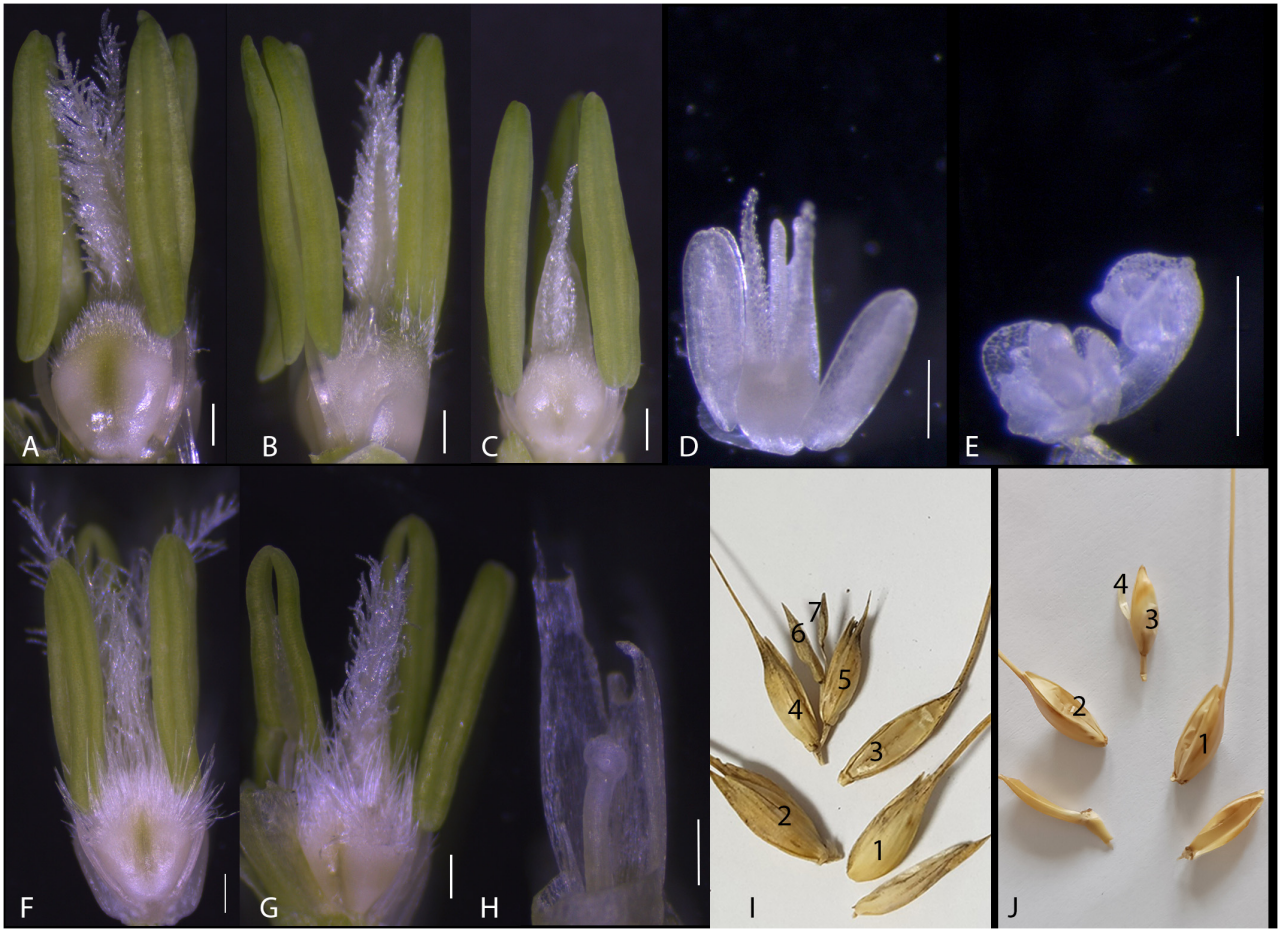
Fertile florets developing in the Latino and MG5323 parental lines. **(A–E)** An average of six fertile florets develop within the central spikelets in the Latino parental line. **(F–H)** An average of four fertile florets develop within the spikelet of the MG5323 parental line. **(I)** Overview of a mature Latino’s spikelet; the seventh floret is always unfertile. **(J)** Overview of a mature MG5323’s spikelet; the fourth floret is always unfertile. Scale bars indicate 200 micron.

The durum wheat *cv* Latino and the emmer accession MG5323, parent genotypes of the RIL population Latino x MG5323, develop spikes with different characteristics (**Table 1** and **Supplementary Table 2**). Indeed, two kernels per spikelet differentiate in MG5323 while five to six kernels are filled in most of the Latino’s spikelets. To further dissect spike differentiation in the parental genotypes, we analyzed spikelet development at selected time points, from the double ridge until the yellow anther stage. Based on our results, we concluded that the difference in the final grain number is strictly associated to the development of the number of florets per spikelet, at least in the central spikelets of the main tillers, with six to eight floret meristems in Latino and strictly 4 in MG5323. Subsequently, floral organ differentiation, fertilization and seed development proceed similarly in the two genotypes, with the abortion of two florets per spikelet in both genotypes (**Figure 1**).

**Table 1:** Summary table of multi-environment values for parental lines and RILs, and variance components for all considered phenotypic traits.

Trait	Latino	MG5323	*P*-value*	RIL max	RIL mean	RIL min	σ^2^_G_	σ^2^_GxE_	σ^2^_ε_	CV%	*H^2^*
SPW	3.4	2	**	5	2.5	0.6	0.11	0.06	0.21	18.44	0.77
FRT	5.6	3.2	***	7.6	4.4	3	0.2	0.03	0.12	7.815	0.92
HD	35.1	48.4	***	61	42.2	31	13.24	2.19	4.59	0.07	0.93
NFRT	4.9	2.8	***	6.6	3.8	2	0.12	0.03	0.11	8.75	0.88
NSPK	20	16.6	***	26.8	17.8	8.8	3.84	0.45	2.09	8.13	0.93
SD	2.5	2.4	ns	4.3	2.5	1.6	0.06	0.01	0.05	9.18	0.90
SPK	20.1	17.9	**	27.6	18.5	10.7	3.88	0.44	1.64	6.9	0.94
SPL	8	7.4	*	10.5	7.5	4.1	0.42	0.09	0.41	8.51	0.88

*Level of significance at P<0.05: ** P<0.01, *** P<0.001 and non-significant (ns). CV, coefficient of variation (%); σ^2^G, genotypic variance; σ^2^GxE, variance of genotype by environment interactions; σ^2^ε, residual variance, H2, broad sense heritability. Trait acronyms: SPW stands for spike weight, FRT for total floret number, HD for heading date, NFRT for net floret number, NSPK for net spikelet number, SD for spike density, SPK for total spikelet number, and SPL for spike length.

The measurement of FRT, NFRT, SPK, and NSPK were performed using both fresh and dry-collected spike in the four environments (F19, F20, F21 and P20) for the parental genotypes and the entire RIL population. Since a strong positive and significant (*p*< 0.01) correlation was obtained between dry and fresh corresponding phenotypic data (FRT_d vs FRT_f, NFRT_f vs NFRT_d, SPK_d vs SPK_f, and NSPK_d vs NSPK_f) (**Supplementary Figure 1**), only data scored on fresh spikes (Waddington scale 9.5) were used for the following analyses.

Descriptive statistics assessed on the parental lines showed significant contrasting phenotypes for most of the traits based on both single (F19, F20, F21 and P20) and multi-environment (MEnv) data (**Supplementary Table 2** and **Table 1**, respectively). Indeed, a *t*-test between the means of each trait calculated for the parental lines showed a significant difference for HD, FRT and NFRT in all single environments (**Supplementary Table 2**). SPW was significantly different in F20 and F21, SPK was significant in F21 only, while NSPK was significant in all environments, with the exception of P20. The *t*-test analysis performed using multi-environment data showed statistically significant differences for all the evaluated traits among the parental lines, except for the trait SD (**Table 1**). In general, the comparison among parents revealed, as expected, that the emmer wheat MG5323 was later in flowering, had fewer florets, lower spike weight, and shorter spike length.

**Figure 2:**
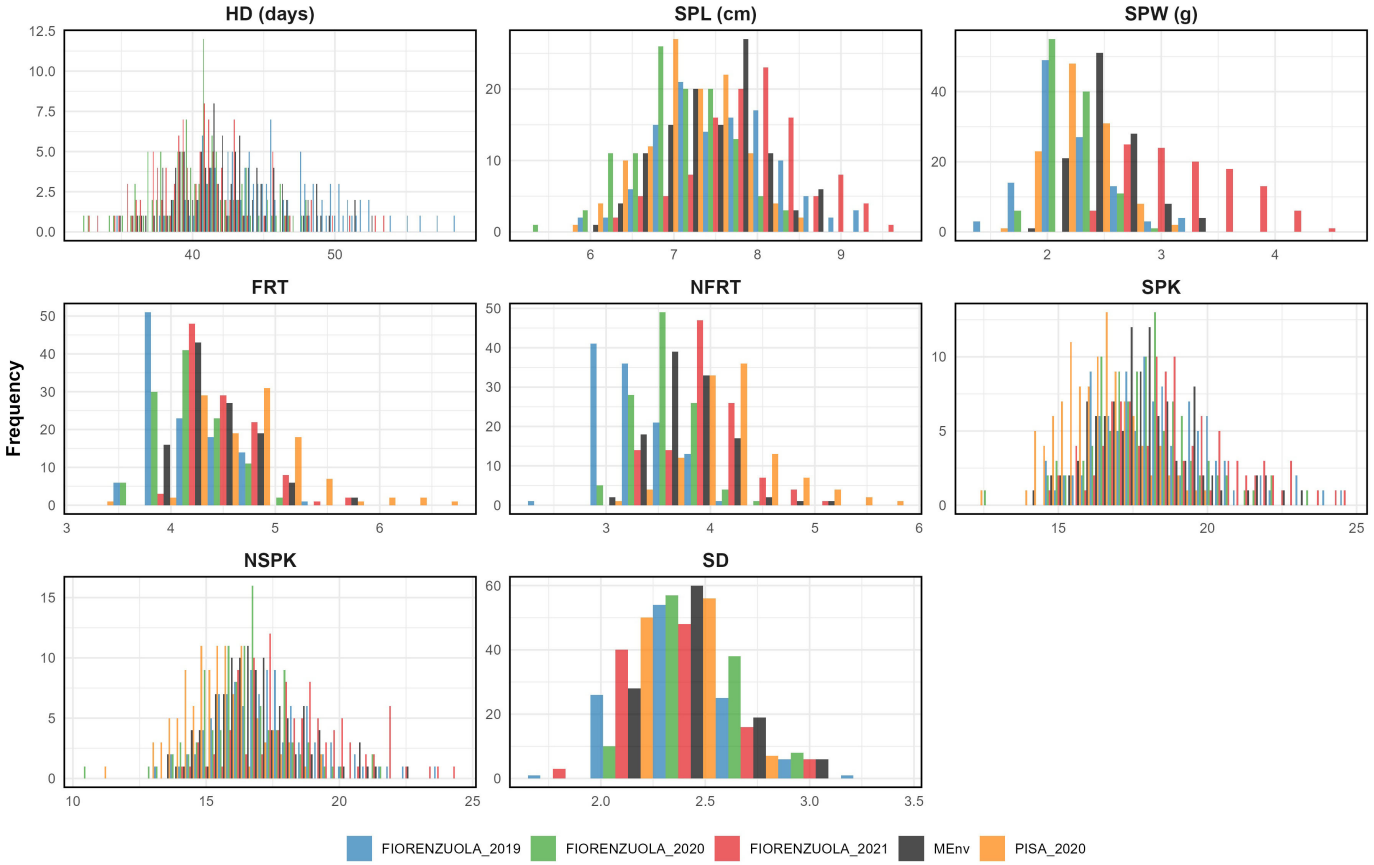
The frequency distribution for all traits of data collected from single and multi-environment BLUPs (light blue for F19, green for F20, red for F21, orange for P20 and black for MEnv, multi-environments data).

The frequency distribution of the RILs differed considerably across locations. Values for skewness and kurtosis were in the range of ±2 for all traits in all environments, suggesting a polygenic inheritance (**Supplementary Table 3** and **Figure 2**). Although the Shapiro-Wilcoxson test did not support the normality of the distributions, the range of skewness and kurtosis supported the interpretation of the traits being in an acceptable range of normality (Webster and Oliver, 2007; Hair et al., 2022). Transgressive segregation was observed in both directions for all the traits analyzed (**Table 1**). As a result, some RILs had higher or lower floret number per spikelet, spikelet number per spike, spike weight, spike length and spike density than those of either of the parents.

For each trait, phenotypic data from RILs were subjected to ANOVA for each single environment and for multi-environment. The ANOVA confirmed significant differences between RILs for all traits in each environment (**Supplementary Table 4**). Estimated variances of the replicates were, in some cases, also statistically significantly different for all the traits in each environment, but the highest variance was always contributed by the genotype effect. As observed, there was a large environmental effect on trait values (*p*< 0.001), which indicated that the differences in the environments considered for this study might allow to identify possible GxE effect on the target traits (**Supplementary Table 5**). For this reason, we computed and used BLUP values in QTL mapping for both single environment and multi-environment data analysis.

The partition of phenotypic variance into genetic, environmental, and GxE interaction variances through the linear mixed model (**Table 1**) demonstrated that the genotypic variance was greater than both GxE interaction and environmental variance except for NFRT and SPW. Due to high genotypic variation a high broad sense heritability was estimated for all traits, which ranged from 77% (SPW) to 94% (SPK) (**Table 1**). The residual coefficient of variation (CV, %) presented magnitudes that ranged from 5.07 to 18.44 for HD and SPW, respectively (**Table 1**).

**Figure 3:**
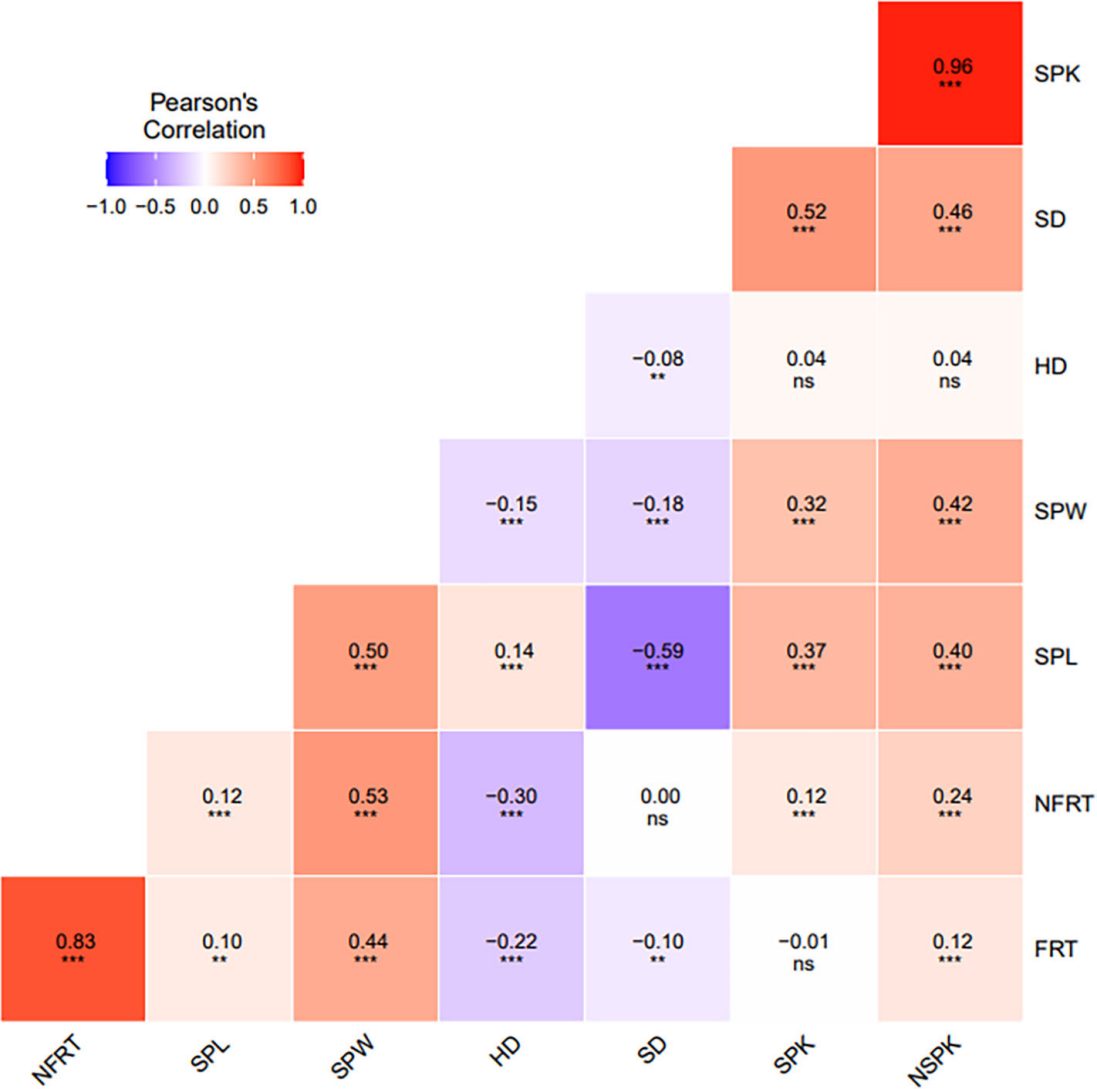
Pearson’s correlation analysis between all spike and floret related traits and heading date using multi-environment BLUPs. Level of significance at P <0.05, ** P <0.01, *** P <0.001 and non-significant (ns).

Pearson’s correlation coefficients between traits were computed based on the BLUP for each single environment and for multiple environments. The correlation analysis using multi environment values showed a positive and significant correlation between traits related to florets (FRT vs NFRT: r=0.83, *p*<0.001) and spikelets (SPK and NSPK: r=0.96, *p*<0.001), as well as between SPW and spikelets and florets traits (from 0.32 to 0.53, respectively, *p*<0.001). SPL was positively correlated with SPW (r=0.50, *p*<0.001) and negatively correlated with SD (r=-0.59, *p*<0.001). Significant and negative correlation was detected between HD and SD (r=-0.08, *p*<0.01), florets related traits and SPW (from -0.22 to -0.15, respectively, *p*<0.001), while a positive correlation was observed with SPL (r=0.14, *p*<0.001) (**Figure 3**). HD was uncorrelated with both SPK and NSPK, and negatively correlated with FRT and NFRT. The same trend was observed using values from each single environment (**Supplementary Figure 2**).

To reduce the dimensionality of the dataset and identify the main sources of variation among the traits, Principal Component Analysis (PCA) was implemented on the variables HD, FRT, NFRT, SPK, NSPK, SPL, SPW, and SD. The results showed that the first three principal components (PCs) had eigenvalues greater than one, explaining 84.8% of the total variation in the data (**Supplementary Figure 3**). The first PC, accounting for 35.5% of the variation, showed a strong correlation with SPK (r=0.96), NSPK (r=1), SPW (r=0.54), SD (r=0.38), SPL (r=0.26) and NFRT (r=0.57). The second PC explained 28.4% of the variation and had strong positive relationships with FRT (r=0.99), NFRT (r=0.94), HD (r=0.26) and SPW (r=0.16). The third PC accounted for 20.9% of the variation and was positively related to SPL (r=0.65) and SD (r=0.67). This analysis further consolidated the results obtained in the Pearson’s correlation analysis.

### Quantitative trait loci analysis

A total of 94 QTLs were mapped, corresponding to 46 different genomic regions. Seventy-four QTLs out of 94 were identified based on BLUPs from single-environment data and 20 QTLs based on BLUPs from multi-environments (**Table 2**). The highest number of QTLs was identified for NSPK and SPK (18 for each), followed by FRT (15), and SPL (13). Considering all the QTLs identified, the proportion of the phenotypic variation explained ranged from 5.9 to 33%. Five QTLs (QNFRT.gb_2A, QSPK.gb_2A, QNSPK.gb_2A, QNSPK.gb_5A and QSPK.gb_7A) were consistently detected using data from both single environment and from multi environment analysis. In the following paragraphs, we discuss QTL results obtained for each phenotypic trait.

**Table 2:** QTLs detected in the MG5323 × Latino RIL segregating population for traits under study.

QTL_Name	Trait	ENV	Chr	cM	LOD	R2	CI_Statrt (cM)	CI_End (cM)	Additive effect
QFRT.gb_1B.1	FRT	F20	chr1B	58.1	3.8	8.2	49.0	67.2	-0.09
QFRT.gb_1B.2	FRT	MEnv	chr1B	125.1	4.5	11.4	118.6	131.6	-0.13
QFRT.gb_2A.1	FRT	MEnv	chr2A	69	6.3	16.5	64.5	73.5	-0.16
QFRT.gb_2A.1	FRT	F19	chr2A	70.3	7.0	18.0	66.2	74.4	-0.16
QFRT.gb_2A.1	FRT	F20	chr2A	70.3	7.7	18.0	66.2	74.4	-0.15
QFRT.gb_2A.1	FRT	P20	chr2A	74.1	6.3	19.5	70.3	77.9	-0.24
QFRT.gb_2A.2	FRT	F21	chr2A	86.6	4.2	11.8	80.3	92.9	-0.14
QFRT.gb_3A	FRT	F20	chr3A	165.7	5.1	11.4	159.2	172.2	-0.11
QFRT.gb_3B	FRT	F21	chr3B	57.3	5.2	14.8	52.3	62.3	-0.15
QFRT.gb_4A.1	FRT	F19	chr4A	0	6.5	16.5	0.0	4.5	-0.14
QFRT.gb_4A.1	FRT	MEnv	chr4A	0	4.6	11.6	0.0	6.4	-0.13
QFRT.gb_4A.2	FRT	F20	chr4A	68.2	5.5	12.2	62.1	74.3	-0.11
QFRT.gb_4B	FRT	F21	chr4B	32.2	4.2	11.8	25.9	38.5	-0.13
QFRT.gb_5A	FRT	F19	chr5A	136.9	3.8	9.0	128.7	145.1	-0.10
QFRT.gb_6A	FRT	P20	chr6A	99.9	3.9	11.5	93.4	106.4	-0.17
QNFRT.gb_2A	NFRT	F21	chr2A	68.4	4.5	17.3	64.1	72.7	-0.16
QNFRT.gb_2A	NFRT	F19	chr2A	70.3	5.9	16.2	65.7	74.9	-0.13
QNFRT.gb_2A	NFRT	F20	chr2A	70.3	6.1	19.9	66.6	74.0	-0.13
QNFRT.gb_2A	NFRT	P20	chr2A	74.1	5.2	15.3	69.3	78.9	-0.19
QNFRT.gb_2A	NFRT	MEnv	chr2A	74.1	6.4	20.3	70.4	77.8	-0.16
QNFRT.gb_4A.1	NFRT	F19	chr4A	0	4.7	12.4	0.0	6.0	-0.11
QNFRT.gb_4A.2	NFRT	F20	chr4A	68.9	3.5	10.8	62.1	75.7	-0.09
QNFRT.gb_5A	NFRT	F19	chr5A	136.9	4.1	10.6	129.9	143.9	-0.10
QNFRT.gb_6A	NFRT	MEnv	chr6A	99.9	3.1	9.3	91.9	107.9	-0.10
QNFRT.gb_6A	NFRT	P20	chr6A	99.9	5.4	16.1	95.3	104.5	-0.18
QNSPK.gb_2A	NSPK	F20	chr2A	111.2	4.5	11.2	104.6	117.8	-0.61
QNSPK.gb_2A	NSPK	F21	chr2A	111.2	4.3	11.4	104.7	117.7	-0.70
QNSPK.gb_2A	NSPK	F19	chr2A	111.2	7.4	18.1	107.1	115.3	-0.85
QNSPK.gb_2A	NSPK	MEnv	chr2A	111.2	9.2	20.4	107.6	114.8	-0.86
QNSPK.gb_2A	NSPK	P20	chr2A	111.2	10.9	22.8	108.0	114.4	-0.88
QNSPK.gb_4A	NSPK	F21	chr4A	102	5.3	17.1	97.7	106.3	0.29
QNSPK.gb_5A	NSPK	F21	chr5A	161.4	3.6	9.2	153.3	169.5	-0.65
QNSPK.gb_5A	NSPK	P20	chr5A	161.4	6.4	12.2	155.3	167.5	-0.67
QNSPK.gb_5A	NSPK	F19	chr5A	161.4	6.4	15.3	156.5	166.3	-0.80
QNSPK.gb_5A	NSPK	MEnv	chr5A	161.4	7.6	16.2	156.8	166.0	-0.79
QNSPK.gb_5A	NSPK	F20	chr5A	161.4	10.9	31.6	159.1	163.7	-1.05
QNSPK.gb_5B.1	NSPK	P20	chr5B	43.7	4.3	7.8	34.2	53.2	0.56
QNSPK.gb_5B.2	NSPK	F20	chr5B	98.9	3.6	8.8	90.5	107.3	0.56
QNSPK.gb_6B	NSPK	F21	chr6B	78.6	5.7	18.6	74.6	82.6	0.31
QNSPK.gb_7A	NSPK	P20	chr7A	148.1	6.8	13.1	142.4	153.8	-0.69
QNSPK.gb_7A	NSPK	F19	chr7A	148.8	5.3	12.2	142.8	154.8	-0.72
QNSPK.gb_7A	NSPK	F21	chr7A	148.1	6.0	16.3	143.5	152.7	-0.86
QNSPK.gb_7A	NSPK	MEnv	chr7A	148.8	7.1	14.9	143.8	153.8	-0.76
QSD.gb_2A	SD	F20	chr2A	111.2	4.7	12.3	105.2	117.2	-0.08
QSD.gb_4A	SD	F19	chr4A	106.5	3.6	11.7	100.2	112.8	-0.08
QSD.gb_4A	SD	P20	chr4A	109	5.7	17.7	104.8	113.2	-0.07
QSD.gb_5A.1	SD	P20	chr5A	152.8	7.3	23.5	149.6	156.0	-0.08
QSD.gb_5A.2	SD	F19	chr5A	161.4	5.4	18.0	157.3	165.5	-0.10
QSD.gb_5A.2	SD	F20	chr5A	161.4	5.7	21.3	157.9	164.9	-0.11
QSD.gb_5A.2	SD	MEnv	chr5A	160.1	7.6	27.1	158.7	164.1	-0.12
QSD.gb_5A.2	SD	F21	chr5A	161.4	10.9	33.0	159.2	163.6	-0.12
QSPK.gb_2A	SPK	F21	chr2A	111.2	6.2	16.6	106.7	115.7	-0.87
QSPK.gb_2A	SPK	F20	chr2A	111.2	7.9	21.7	107.8	114.6	-0.90
QSPK.gb_2A	SPK	MEnv	chr2A	111.2	11.2	26.1	108.4	114.0	-0.98
QSPK.gb_2A	SPK	F19	chr2A	111.2	13.3	30.3	108.8	113.6	-1.12
QSPK.gb_2A	SPK	P20	chr2A	111.2	15.7	30.7	108.8	113.6	-0.97
QSPK.gb_4B	SPK	P20	chr4B	65.6	3.9	5.9	53.0	78.2	0.44
QSPK.gb_5A	SPK	F20	chr5A	161.4	3.5	8.9	153.1	169.7	-0.60
QSPK.gb_5A	SPK	MEnv	chr5A	161.4	4.9	9.9	153.9	168.9	-0.63
QSPK.gb_5A	SPK	P20	chr5A	164.8	5.1	7.8	155.3	174.3	-0.52
QSPK.gb_5A	SPK	F19	chr5A	166.1	4.1	7.6	156.3	175.9	-0.58
QSPK.gb_5B	SPK	P20	chr5B	43.7	5.9	9.3	35.7	51.7	0.58
QSPK.gb_7A	SPK	F20	chr7A	148.8	4.4	11.4	142.3	155.3	-0.67
QSPK.gb_7A	SPK	P20	chr7A	148.8	9.3	15.6	144.1	153.5	-0.72
QSPK.gb_7A	SPK	F19	chr7A	148.8	8.5	17.4	144.5	153.1	-0.87
QSPK.gb_7A	SPK	MEnv	chr7A	148.8	8.0	17.4	144.5	153.1	-0.83
QSPK.gb_7A	SPK	F21	chr7A	148.1	9.1	25.9	145.2	151.0	-1.07
QSPK.gb_7B	SPK	F19	chr7B	48.1	4.3	8.1	39.0	57.2	0.58
QSPK.gb_7B	SPK	F21	chr7B	48.1	3.7	9.4	40.2	56.0	0.65
QSPL.gb_2A	SPL	P20	chr2A	121.8	4.9	11.8	115.5	128.1	-0.19
QSPL.gb_3B	SPL	P20	chr3B	109	3.6	8.3	100.1	117.9	-0.17
QSPL.gb_3B	SPL	MEnv	chr3B	109	4.1	11.6	102.6	115.4	-0.21
QSPL.gb_3B	SPL	F19	chr3B	109	4.7	14.7	104.0	114.0	-0.28
QSPL.gb_4A.1	SPL	F20	chr4A	41.9	3.9	11.6	35.5	48.3	0.21
QSPL.gb_4A.1	SPL	P20	chr4A	41.9	6.8	17.1	37.6	46.2	0.25
QSPL.gb_4A.2	SPL	MEnv	chr4A	102	5.6	16.4	97.5	106.5	0.25
QSPL.gb_4A.2	SPL	F19	chr4A	105.2	5.1	16.0	100.6	109.8	0.29
QSPL.gb_5B.1	SPL	P20	chr5B	43.7	5.7	13.9	38.4	49.0	0.23
QSPL.gb_5B.2	SPL	F20	chr5B	98.9	3.7	10.7	92.0	105.8	0.19
QSPL.gb_6B	SPL	F19	chr6B	78	4.1	12.8	72.2	83.8	-0.26
QSPL.gb_6B	SPL	MEnv	chr6B	78.6	6.8	20.4	75.0	82.2	-0.28
QSPL.gb_6B	SPL	F20	chr6B	86.1	3.6	10.5	79.1	93.1	-0.19
QSPW.gb_1A	SPW	MEnv	chr1A	112.4	3.6	12.4	106.4	118.4	-0.10
QSPW.gb_2A	SPW	P20	chr2A	112.5	3.7	14.2	107.3	117.7	-0.10
QSPW.gb_6B	SPW	MEnv	chr6B	67.1	5.1	17.8	62.9	71.3	-0.13
QHD.gb_2A	HD	MEnv	chr2A	39	3.7	9.0	30.8	47.2	1.07
QHD.gb_2A	HD	F20	chr2A	39	4.1	10.6	32.0	46.0	1.09
QHD.gb_2B	HD	F21	chr2B	46.4	6.0	22.2	43.1	49.7	1.81
QHD.gb_2B	HD	F20	chr2B	46.4	8.1	22.5	43.1	49.7	1.61
QHD.gb_2B	HD	MEnv	chr2B	46.4	9.5	26.2	43.6	49.2	1.86
QHD.gb_2B	HD	F19	chr2B	46.4	8.7	27.5	43.7	49.1	2.40
QHD.gb_7B	HD	F19	chr7B	39.9	3.7	10.4	32.7	47.1	1.42
QHD.gb_7B	HD	F20	chr7B	54.5	3.9	10.0	47.1	61.9	1.04
QHD.gb_7B	HD	MEnv	chr7B	54.5	4.1	10.1	47.2	61.8	1.12

Chromosomes (Chr), QTL peak position (cM), LOD scores, percentages of phenotypic variance explained (R2, %), the confidence interval (CI) and estimated additive effects were also provided. The + or − signs in the last column indicate the positive or negative additive effect of the parental line MG5323.

Trait acronyms: FRT stands for total floret number, NFRT for net floret number, SPK for total spikelet number, NSPK for net spikelet number, SPW for spike weight, SD for spike density, SPL for spike length and HD for heading date, Environments abbreviations: F19, F20 and F21 for Fiorenzuola d’Arda field experiment, P20 for Pisa field trial in 2020 and MEnv for multi-environment data analysis.

### Floret number per spikelet

Twenty-five QTLs associated with both FRT and NFRT were identified (**Table 2**). They were located on chromosomes 1B, 2A, 3A, 3B, 4A, 4B, 5A, and 6A. Of these, the QTL region detected on chromosome 2A (named QFRT.gb_2A.1 and QNFRT.gb_2A) was stably detected in each single environment, as well as in the multi-environment analysis, and the phenotypic explained variation varied from 15.3% to 20.3%. Two regions were identified on chromosome 4A for both FRT and NFRT and the highest phenotypic variance (16.5%) was detected by QFRT.gb_4A.1 using data from F19. Six environment-specific regions were identified for FRT (on chromosomes 1B, 2A, 3A, 3B and 4B) while common regions between traits (FRT and NFRT) were detected on chromosomes 5A and 6A. The additive effect showed that Latino contributed alleles determining a higher number of florets at all loci.

### Spikelet number per spike

A total of 36 QTLs for both SPK and NSPK were identified (**Table 2**). Of these, the QTLs detected on chromosomes 2A, 5A and 7A were stable and consistently detected in all four single environments and in the multi-environment analysis. The first QTL region on chromosome 2A (QSPK.gb_2A and QNSPK.gb_2A) showed a phenotypic variance ranging from 11.2% to 30.7%. The second stable region detected on chromosomes 5A (QSPK.gb_5A and QNSPK.gb_5A) explained a phenotypic variance ranging from 7.6% to 31.6%, while the last one on chromosome 7A (QSPK.gb_7A and QNSPK.gb_7A) showed a phenotypic variance up to 26%. For all these stable QTLs (on chromosomes 2A, 5A and 7A) the additive effect values showed that Latino contributed with alleles responsible for the higher spikelet number.

### Spike weight

Three QTLs associated to SPW were identified on chromosomes 1A, 2A, and 6B (**Table 2**). QSPW.gb_1A and QSPW.gb_6B were detected based on multi-environment data while QSPW.gb_2A using data from P20 only. The phenotypic variance ranged from 12.4% to 17.8% and the additive effect revealed that Latino positively contributed to SPW in terms of higher spike weights.

### Spike length

Thirteen QTLs for SPL were detected on chromosomes 2A, 3B, 4A, 5B, and 6B. The phenotypic variance of QTLs reported for this trait ranged from 8.3 to 20.4% (**Table 2**). QSPL.gb_3B and QSPL.gb_6B were detected in three environments, whereas the two distinct QTLs identified on chromosomes 4A (QSPL.gb_4A.1 and QSPL.gb_4A.2) were obtained using data from two environments each. Both parents contributed the allele with positive effect on SPL: MG5323 contributed to QTLs identified on chromosomes 4A and 5B, while Latino to the regions on chromosomes 2A, 3B and 6B.

### Spike density

The SD was obtained from the ratio of SPK and SPL. Eight QTLs, on chromosomes 2A, 4A, and 5A, were identified (**Table 2**). Of these, QSD.gb_5A.2 (F21) represented the major QTL with a phenotypic variance explained of 33%, and a LOD value of 10.9. The additive effect value indicated Latino as the positive contributor of all QTLs for SD.

### Heading date

Nine regions associated to HD were identified on chromosomes 2A, 2B, and 7B. The QHD.gb_2B was consistent in each environment tested and in the multi-environment analysis, with a maximum phenotypic variance of 27.5% found in F19. The QTL on chromosome 7B (QHD.gb_7B) explaining a maximum of 10.4% phenotypic variance, was detected in F19, F20 and in the multi-environment analysis (**Table 2**). As shown from the values of additive effects, MG5323 contributed with the late HD allele for all QTLs identified.

### Physical inspection of regions with overlapping QTLs

The confidence intervals of the 94 individual QTLs were determined to assess if they span fully or partly overlapping regions on the reference durum wheat genome assembly.

Based on co-location of the confidence interval of each QTL (**Supplementary Table 6**), 17 QTL groups were identified, 7 of these groups contained QTLs for multiple traits. More in detail, group3_chr2A and group11_chr5A hosted QTLs for spikelet number per spike and spike density; group9_chr4A and group15_chr6B QTLs for spikelet number per spike, spike length and other spike related traits, group12_chr5B and group13_chr5B included QTLs related to spikelet number per spike and SPL and group17_chr7B with QTL for HD and SPK (**Table 3** and **Supplementary Table 6**). Interestingly, no QTL group included QTLs for both floret number per spikelet and spike-related traits or heading date.

**Table 3:** Group of QTLs and their corresponding physical interval.

Group					Best QTL		Known genes	Tetraploid QTL/MTA
N^0^	Chr	Start (Mbp)	End (Mbp)	Traits	Start (Mbp)	End (Mbp)		
1	2A	29.53	48.26	**HD**	29.53	48.26	*Ppd-A1^48,50^*	GY^1,28^, HD^1,2,28,58,59^, KNM^3^, KWS^4^, TKW^4,28^, TKW^3^, KA^28^, KL^28^, KW^28^, DM^58^, SLNS^58^, SPL^58^, DB^58^, DF^58^, KS^59^, KWL^59^
2	2A	126.52	569.87	**NFRT**, FRT	191	569.87	*FUL-A2^40^*, *FUL-A3^40^*, *TaCwi-A1^45^*, *HOX2^47^*, *SPL13-2A^34^*	GY^6^, KNS^7^, KL^23^, KLW^23,60^, KS^60^, TKW^60^, KW^23^
3	2A	685.56	732.72	**SPK**, NSPK, SPL, SD, SPW	685.56	709.55	*LEAFY-like-A^52^*	SD^8,9^, SNP^6^, TKW^10,58^
4	2B	34.74	52.57	**HD**	38.61	52.57	*Ppd-B1^48,50^* (56.29 Mbp)	SPL^58^, DB^58^, DF^58^, HD^58,59^, DM^58^, KP^59^, KS^59,61^, KWL^61^, KL^23^
5	3B	663.04	740.01	**SPL**	679.08	735.24	*AGL33-B^54^*	TKW^58,59^, KA^59^
6	4A	0.26	7.16	**FRT,** NFRT	0.26	7.16	*SVP-A3^41^*	HD^23^
7	4A	39.83	257.26	**SPL**	59.48	143.33	*PHYB-A^31^*	SPL^58^, SLNS^58^, SHI^10^, HD^27^
8	4A	571.16	589.33	**FRT**, NFRT	571.16	586.31	*TB1^33^*	SHI^10^, TKW^10^, HI^27^
9	4A	611.98	645.42	**SD**, SPL, NSPK	626.91	645.42	*CEN4-A^41^*	FLA^12^, GY^6,26^, HD^13^, SW^5,14^, TW^13^, KL^24^, KWL^24^
10	5A	527.52	557.66	**NFRT**, FRT	529.44	557.66	*VRN-A1^51^, AGLG1-A^30,51^, PhyC-A/SEP1-6A/PAP2^49,53^*	KNS^7^, KW^24^
11	5A	560.52	629.68	**SD**, SPK, NSPK	586.61	620.90	*Q^56,57^*	DA^12^, FLA^12^, FRT^15^, HD^16,23^, KN/CHAFF^12^, KWP^15^, PHY^12^, SNP^12^, TKW^25,26^
12	5B	59.23	405.79	**SPL**, NSPK, SPK	59.23	335.61	*TaAGL31-B^54^*, *TaFDL-B6^38^*, *CEN5-B^41^*	KNS^7^, SNP^6^
13	5B	523.7	545.14	**SPL**, NSPK	523.7	542.9		DM^58^, DB^58^, DF^58^, HD^58^, GY^6^, HI^14^, KWS^17,18^
14	6A	530.74	571.7	**NFRT**, FRT	554.42	568.42	*SVP-A1^41^*, *KRP1-A^29^*	TKW^7^
15	6B	263.24	629.98	**SPL**, SPW, NSPK	500.16	551.41	*TaGW2-B^55^*, *TaAGL6-B1^54^*, *CONSTANS-B2* ^43^, *GS1a-6B^46^*, *SVP-B1^41^*, *KRP1-B^29^*	KNS^19^, KNSL^15^, KWS^20^, SLNS^12^, SNP^12^, TKW^11,21,28^, KA^24^, KL^24,28^, KP^24^, KWL^24^, HI^27^
16	7A	663.05	675.55	**SPK,** NSPK,	664.36	673.52	*WAPO-A1^37^*	
17	7B	64.9	414.55	**HD**, SPK	64.9	115.17	*TaSus1-7B^35^*, *KRP-B2^29^*, *SVP-B2^41^*, *TaGASR-B^32^*, *CONSTANS-B1^43^*, *NF-YB-B1^39^*, *TaTGW-7B^36^*, *SEP3-1B^41^*, *TaCYP78A16-B^42^*, *Ehd3-B^44^*	FRT^5^, HD^13^, KWS^22^, TKW^22^, TW^13^

The best QTL (in bold), the wheat known genes/QTL (associated with yield, phenology and domestication) co-located in the physical interval are also reported.

^1^ (Milner et al., 2016), ^2^ (Maccaferri et al., 2008), ^3^ (Graziani et al., 2014), ^4^ (Blanco et al., 2012), ^5^ (Alemu et al., 2020), ^6^ (Mengistu et al., 2015), ^7^ (Kidane et al., 2017), ^8^ (Thanh et al., 2013), ^9^ (Faris et al., 2014b), ^10^ (Peleg et al., 2011), ^11^ (Tzarfati et al., 2014), ^12^ (Giunta et al., 2018), ^13^ (Maccaferri et al., 2011), ^14^ (Peleg et al., 2009), ^15^ (Roncallo et al., 2017), ^16^ (Maccaferri et al., 2014), ^17^ (Faris et al., 2014a), ^18^ (Golabadi et al., 2011), ^19^ (Peng et al., 2003), ^20^ (Marcotuli et al., 2017), ^21^ (Elouafi and Nachit, 2004), ^22^ (Mangini et al., 2018), ^23^ (Peters Haugrud et al., 2023), ^24^ (Desiderio et al., 2019), ^25^ (Fatiukha et al., 2020), ^26^ (Giancaspro et al., 2019), ^27^ (Negisho et al., 2022), ^28^ (Mangini et al., 2021), ^29^ (Ajadi et al., 2020), ^30^ (Chen and Dubcovsky, 2012 (Chen and Dubcovsky, 2012), ^31^ (Chen et al., 2014) ^32^ (Cheng et al., 2019), ^33^ (Dixon et al., 2018), ^34^ (Gupta et al., 2023), ^35^ (Hou et al., 2014), ^36^ (Hu et al., 2016), ^37^ (Kuzay et al., 2022), ^38^ (Li and Dubcovsky, 2008), ^39^ (Li et al., 2011), ^40^ (Li et al., 2019), ^41^ (Li et al., 2021), ^42^ (Linnan et al., 2018), ^43^ (Shaw et al., 2020), ^44^ (Matsubara et al., 2011), ^45^ (Ma et al., 2012), ^46^ (Pascual et al., 2023), ^47^ (Sakuma et al., 2019), ^48^ (Takenaka and Kawahara, 2012), ^49^ (VanGessel et al., 2022), ^50^ (Wilhelm et al., 2009), ^51^ (Yan et al., 2003), ^52^ (Wang et al., 2017a), ^53^ (Wang et al., 2017b), ^54^ (Zhao et al., 2006), ^55^ (Zhang et al., 2018), ^56^ (Zhang et al., 2011), ^57^ (Debernardi et al., 2020), ^58^ (Gesesse et al., 2023), ^59^ (Valladares García et al., 2023), ^60^ (Sesiz, 2023). The acronyms for the known QTL for yield and yield component are: GY, Grain yield; TKW, Thousand kernel weight; HI, Harvest index; KNM, Kernels per square meter; KWS, Grain yield per spike; KA, Kernel area; KL, Kernel length; KW, Kernel width; KNS, Kernels per spike; KWL, Kernel width to length ratio; SNP, Spikes per plant; SD, Spike density; SHI, Spike harvest index; SW, Spike dry matter; TW, Test weight; KN/CHAFF, Kernels number per gram of chaff; KNSL, Kernels per spikelet; SLNS, Spikelets per spike; KP, Kernel perimeter; KS, Kernel shape; SPL, Spike length. Known QTL related to phenological traits are: HD, Heading date; DA, Days to anthesis; DB, Days to booting; DF, Days to flowering; DM, Days o maturity; FLA, Flag leaf appearance.

To define the physical interval of each group, the best QTL (QTL with the highest LOD and R^2^) was selected. In this way, the largest physical regions were detected on chromosome 2A (group2), 4A (group7) and 5B (group12), which spanned for more than 80 Mbp (378.8 Mbp, 83.8 Mbp and 276.3 Mbp, respectively) and included the centromeric regions as reported by Maccaferri et al., 2019 (specifically in Table S22 of Maccaferri et al., 2019). Two groups (6 and 16) identified a smaller region of 10 Mbp (6.89 Mpb and 9.15 Mbp, respectively), 8 groups (1, 3, 4, 8, 9,10,13 and 14) extended for 35 Mbp, and 3 groups spanned for approximately 50 Mbp (5, 15 and 17) (**Table 3** and **Supplementary Table 6**).

### Identification of annotated genes and known QTLs that co-localize with the identified QTLs

The Svevo reference genome was used as common framework to compare the physical position of the 17 QTL groups defined in this study with the map position of genes already described as influencing spike-related traits.

**Figure 4:**
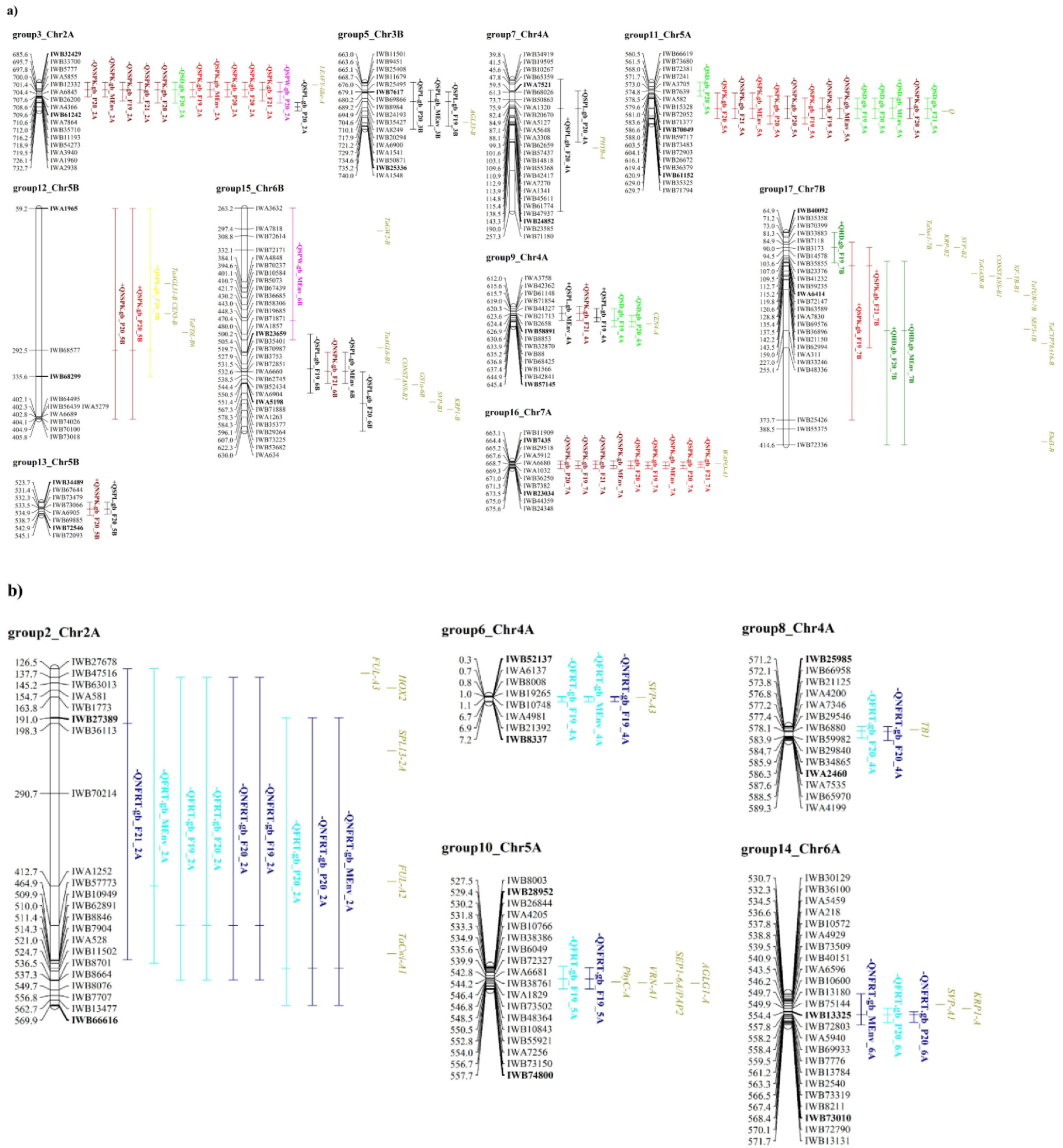
Schematic representation of QTL groups anchored on the durum wheat reference genome. **(A)** QTL groups related to spike traits; **(B)** QTL groups referred to floret traits. Part of the chromosomes are represented by including some markers surrounding the QTL group; SNP marker IDs are on the right, whereas their positions on the durum wheat reference genome are reported in Mbp on the left. The name of flanking markers of the best QTL in each group is in bold. QTL names are according to **Table 2**. The traits are denoted as: FRT, total floret number; NFRT, net floret number; SPK, total spikelet number; NSPK, net spikelet number; SPW, spike weight; SD, spike density; SPL, spike length; HD, heading date. Environments abbreviations: F19, F20 and F21 for Fiorenzuola d’Arda field experiment, P20 for Pisa field trial in 2020 and BL for multi-environment data analysis. Known genes are reported. Complete information of this figure is on **Tables 2** and **3**, **Supplementary Tables 6**, **7**.

Thirty-eight master gene regulators of heading time and flowering time, spike development, spikelet and floral meristem identity co-localized with our QTL groups (**Table 3**; **Figure 4** and **Supplementary Table 6**). For example, *FUL-A2, FUL-A3, TaCwi-A1, HOX2* and *SPL13-2A* genes, known to be involved in inflorescence meristem development (Sakuma et al., 2013; Jiang et al., 2015; Li et al., 2019; Gupta et al., 2023) co-localized with group2_chr2A, which included 9 QTLs for the traits FRT and NFRT (**Table 3**; **Figure 4**, and **Supplementary Table 6**). The group10_chr5A, where few QTLs for floret number per spikelet were identified, was found to co-locate with *PhyC-A*, *VRN-A1*, *AGLG1-A* and *SEP1-6A/PAP2*, which are known to regulate heading time, flowering time, and floral organ identity (Yan et al., 2003; Chen and Dubcovsky, 2012; Wang et al., 2017b). In addition, in group15_chr6B, associated to spike length and spike weight and to spikelet number per spike, six genes were co-localized. In details, here we found *TaGW2-B*, which influences wheat grain width and thousand kernel weight (Qin et al., 2014), and *CONSTANS-B2*, which impacts the wheat photoperiod flowering pathway (Nemoto et al., 2003), and hence, putatively, spike developmental patterns. In addition, the *glutamine synthetase* (*GS*) gene, which was shown to have a positive effect on kernel number per spike, besides on grain protein content and Thousand-Kernel Weight (TKW, Pascual et al., 2023), the *SHORT VEGETATIVE PHASE 1* (*SVP-B1*) gene, a MADS-box gene that plays a critical role in regulating meristem transitions during wheat spike development (Li et al., 2021) and *KRP1-B*, which belongs to the Kip-related protein (KRP) family that regulates cell division and differentiation (Barrôco et al., 2006). Lastly, *TaAGL6-B1*, a member of the *AGAMOUS-LIKE6* (*AGL6*)-like MADS-box gene family, plays a role in regulating floral development and fertility in wheat (Kong et al., 2022).

Known genes controlling heading date, *Ppd-A1* and *Ppd-B1*, mapped within the HD-related QTL groups (group1_chr2A and group4_chr2B, respectively), a finding supporting the different photoperiod requirements of the parental lines Latino and MG5323. Since the *Ppd-A1* and *Ppd-B1* genes co-located with QTL groups related to HD only, we hypothesized that in our mapping population and field conditions phenology variability related to these main regulatory genes did not influence any spike/spikelet-related trait.

Since the spike-related traits analyzed in the present work represent specific yield components, we have compared our data with the tetraploid wheat QTLome (Maccaferri et al., 2019), updated until December 2023. One hundred ten QTLs, previously mapped for yield and yield components or for phenological traits, overlapped with the QTL regions identified in our study (**Table 3**). For example, QHD.gb_2A and QHD.gb_7B may correspond to the QTLs on chromosomes 2A and 7B reported by Maccaferri et al. (2008) and Milner et al. (2016) for HD. Similarly, QSD.gb_2A, QSD.gb_4A, and QSD.gb_5A could correspond to the previously reported QTLs on chromosomes 2A, 4A, and 5A for SD (Thanh et al., 2013; Faris et al., 2014b). In addition, QTLs for SPK and NSPK on chromosomes 5A and 6A were similarly reported in previous studies (Roncallo et al., 2017; Giunta et al., 2018).

Interestingly, no known yield-related gene mapped within group13_chr5B, a QTL that we found associated to spikelet number per spike and spike length. Some previously published QTLs for grain yield (**Table 3**) support that this region is associated to spike-related traits and prompted us investigating new genes associated to these traits.

### Analysis of the transcriptional profiles and polymorphisms to characterize the most interesting genes

To dissect the QTL groups identified in the present paper, we decided to focus our attention on two groups of genes: the list of master genes, involved in heading date, flowering and spike/spikelet development co-localized in in our regions (**Table 3**), and the list of annotated genes in the QTL group13_chr5B (SPL and NSPK), where no previously known yield/spike related genes have been retrieved. We investigated the expression profile, taking advantage of the ExpVip platform, and the nucleotide polymorphisms (SNP) between the MG5323 and the durum wheat reference genomic sequence, to highlight the most interesting genes.

Referring to the master genes, 260 SNPs were identified in 28 genes, out of 38 genes analyzed. The SNP position and the putative effect on the protein properties are reported in **Supplementary Table 8**. The homologous Chinese Spring gene sequences of the above-mentioned genes were assessed for gene transcription on the ExpVIP platform, unfortunately we couldn’t identify any homologous of the *WAPO-A1* gene and no expression data were available for *LEAFY-like-A*, *TB1* and *CONSTANS-B2*; the transcription profile of the remaining 34 genes is reported in **Supplementary Table 9**.

As concerns the genes of QTL group13_chr5B, we considered all the 270 genes annotated in the corresponding Svevo genome region; firstly, the ExpVip platform was interrogated to assess their expression profile. For 26 durum wheat genes, no bread wheat homologous were identified, while for other 46 genes some copy number variation was hypothesized as they matched with 20 bread wheat genes in total only. Based on the expression data, out of the remaining 218 genes (**Supplementary Table 10**), 31 genes showed specific transcription or repression into the wheat inflorescence and/or spike-related tissues (**Supplementary Table 11**), most of which have a role, based on their inferred function, in transcriptional regulation, hormones biosynthesis and signaling. Their inspection for sequence polymorphisms identified 44 SNPs in the coding region of 7 genes and 81 SNPs in the upstream region of 15 genes. The list of the polymorphisms together with their putative effect in case of amino acid change is reported in **Supplementary Table 11**. We have detected missense variants in the following genes: *TRITD5Bv1G184230*, *TRITD5Bv1G185330 TRITD5Bv1G186880, TRITD5Bv1G186910, TRITD5Bv1G188610* and *TRITD5Bv1G188770*, the most polymorphic gene. In all analyzed cases, a moderate effect is expected on the protein structure with the exception of a strong effect due to the change of a stop codon into a lysin codon in the *TRITD5Bv1G185330* coding for a cytochrome P450 factor.

## Discussion

Improving grain yield is a common desirable trait in all cereal crop species, however its complexity has challenged scientists and breeders for decades. Grain yield is mainly influenced by grain size and grain number, the last being more plastic (Sadras and Slafer, 2012), highly influenced by the environment (Mohammadi et al., 2011; Wu et al., 2012; Philipp et al., 2018) but at the same time has shown the highest genetic potential in improving yield in wheat (Shearman et al., 2005; Acreche et al., 2008; Álvaro et al., 2008). The total number of grains is significantly affected by the number of spikelets per spike and by the dynamics of floret generation/degeneration ending up in a certain number of fertile florets per spikelet (Ma et al., 2007; González et al., 2011; Sreenivasulu and Schnurbusch, 2012).

Hundreds of QTLs have been identified for grain number in durum wheat, most of which related to the total number of kernels per spike, the number of spikelets per spike and the number of kernels per spikelet (for a review Arriagada et al., 2020), disregarding the number of fertile florets per spikelet. In this work we performed a QTL mapping of the spike architecture-related traits, taking advantage of the structural differences existing between the spikes of the selected parental lines which specifically differ for the number of florets developing within the spikelet. In particular, we have observed the formation of up to three fertile florets per spikelet in MG5323 and up to six in Latino. In these genotypes, the diversity in seed number/spikelet is therefore a consequence of the differentiation of a different number of florets per spikelet.

Statistics analyses on the phenotypic data of the parental lines and of the RILs confirmed the usefulness of the selected genetic material for the target traits: i) parental lines showed statistically significant differences for all traits, except for SD; ii) the genotype, environment, and GxE interactions had a highly significant effect on the phenotypic variation; iii) a high broad-sense heritability was calculated indicating that the observed phenotypic variation was mainly due to heritable genetic differences among the RILs. All these results are in line with data already published on wheat (Zhang et al., 2017; Kuzay et al., 2019).

### Distinct genomic regions determine spike architecture and floret number in the Latino x MG5323 population

The different yield components analyzed in the present work showed a significant correlation confirming previous knowledge (Serrago et al., 2008; González et al., 2014; Guo et al., 2015). Notably, correlation among floret number (FRT and NFRT) and spikelet number (SPK, NSPK) was significant but low, suggesting that they are under the control of different regulatory factors. Moreover, the higher correlation of floret number traits (FRT, NFRT) than spikelet number (SPK, NSPK) traits with spike weight should be noted. Indeed, the number of fertile florets presented a significant positive correlation with yield related traits at maturity under a wide range of conditions in previous publications (e.g. González et al., 2003, 2005; Zhu et al., 2019), corroborating our approach. The negative correlation between HD and FRT, NFRT and SPW is in line with previous observations showing that in drought prone environments, such as Italy, late heading genotypes usually have lower grain yields than early heading types (Bort et al., 2005; Sobhaninan et al., 2019; Mulugeta et al., 2023; Peters Haugrud et al., 2023); therefore modern high-yielding varieties tend to have earlier heading dates than older varieties, with earliness accompanied by an increase in grain filling length (De Vita et al., 2007; Bassi and Nachit, 2019).

A total of 94 QTLs were detected using data from both single environment and multiple environments analyses. Based on the physical position of the 94 detected QTLs, 17 distinct groups (listed in **Table 3**) were identified. Overall, the traits referred to spike architecture (SPK, NSPK, SPL, SPW) and the secondary trait derived from them, such as spike density (SD=SPK/SPL) often co-localized in the same genomic regions. As an example, group3_chr2A includes QTLs for SPK, NSPK, SPL, SD, and SPW, meaning that spike architecture may be dissected into less complex traits and hence regulated by common genetic determinants (Cheng et al., 2017). Consequently, it would be reasonable to associate spike architecture phenotyping with a trait characterized by an easier phenotyping strategy and a high heritability, such as spike length.

Looking at QTL mapping positions, it is noteworthy that loci associated to floret number (FRT, NFRT) and spikelet number (SPK, NSPKL) were never overlapping, which suggests that floret and spikelet number might be subject to distinct genetic control mechanisms. This observation is reinforced by the dynamic of floret and spike development in wheat, where the spikelet primordia and the floret primordia phases are well timely distinguished. The first spans the meristem developmental period, from the double ridge till the terminal spikelet stage, when the final number of spikelets is set, while during the second, also called stem elongation phase, lasting until anthesis, floret primordia differentiate then turning into fertile florets or abort (González et al., 2011). The data here presented suggest that the genetic network controlling the two phases, and hence floret number and spikelet number, are distinguished.

### Identification of two QTLs that overcome the trade-off between grain yield components

To assess the novelty of our results we compared the physical positions of groups identified in this study with durum wheat QTLs from previous studies (from both linkage and association mapping). Most of the QTLs identified in this work fell within regions previously identified for yield-related traits, despite different genetic backgrounds and experimental conditions. Nonetheless, this study allowed incrementing the number of traits associated to each of the co-locating QTL.

Considering the groups identified as associated to spike related traits (group 3, 5, 7, 9, 11, 12, 13, 15, 16, 17), overlaps with known regions were identified showing that QTL groups that we found associated to spike related traits co-localized with known QTLs previously published as associated not only to spike morphology (spike density, spikelets per spike) but also to TKW, GY, spike dry matter, kernel per spike, and seed morphology. The same can be observed for QTL groups associated to floret traits identified in the present work, showing that the traits analyzed, even though describing the early events of spike development, i.e. the setting of the number of spikelets per spike and the number of fertile floret at anthesis, are strongly related to grain yield.

Finally, we compared our results with the 105 QTLs reported in Valladares García et al. (2023) for kernel size components (length, width, perimeter, and area), kernel shape (width-to-length ratio and form coefficient), and their relationships with kernel weight because the same segregant population was used. The comparison revealed 14 shared genomic regions (**Supplementary Table 7**), among these, we specifically focused on the physical region where QTL for FRT/NFRT and SPK/NSPK were detected, and co-location was supported by inter-peak distances of less than 20 Mbp. Based on these assumptions, five interesting regions, were considered and described below.

In the first region (chromosome 2A, from to 691,356,868 - 700,038,768 bp) where QTLs for spikelet number per spike and spike density together with QTLs for seed size were identified. The analysis of additive effect revealed that RILs carrying the allele from the *cv* Latino had longer and heavier spikes, but smaller seeds (perimeter, P, area, A). The same trend was detected on chromosome 5A (from 616,063,915 to 603,477,060) where QTLs associated to SPK, SD and seed weight were identified. Also, in this genomic region, lines carrying alleles from the durum wheat parent had spike with more spikelets, a higher spike density, but lighter seeds (TKW). Finally, the region detected on chromosome 4B (from 25,926,927 to 41,570,898) where QTLs for FRT, seed weight and width were identified, revealed that RILs with the allele from the emmer parent had larger and heavier seeds but fewer flowers in the spikelet. These three regions showed the trade-off of wheat grain number with negative phenotypic relationships between TKW and grain number components (e.g. Foulkes et al., 2011; Molero et al., 2019 and references cited therein).

Two very interesting regions with evidence against this expected trade-off between yield components were identified. The first one was detected on chromosome 4A (from 620,511,152 to 636,822,157 for traits SD, SL, NSPK and seed perimeter). The RILs carrying the emmer parent allele had longer and less dense spikes, with a greater number of spikelets, but also larger seed size (P). The second one was identified on chromosome 4B (from 521,547,269 to 528,901,924 for traits SPK, TKW, seed area, length and perimeter): the associated alleles determine lines with more spikelets, but also larger, longer and heavier seeds.

For these two regions, two possible genes could be proposed as playing a pivotal role: *CENTRORADIALIS 4* (*CEN4-A*; **Supplementary Table 8**) and *BIG GRAIN PROTEIN 1* (*BG1*) for 4A and 4B, respectively (Valladares García et al., 2023).

The genome sequence comparison between MG5323 and the durum wheat reference genome highlighted only polymorphisms in the upstream gene sequences for both genes (4 for *CEN4-A* and 1 for *BG1*) and a future detailed analysis will be required to understand their possible role. Anyway, our findings demonstrate that these two regions overcome the trade-off between TKW/grain size and grain number, leading us to conclude that these haplotypes and their functional markers could be utilized in marker-assisted selection for breeding high-yielding varieties.

### Comparative gene sequence analysis for known genes among regulators of wheat phenology and inflorescence development

The recent availability of different genome sequences and expression datasets is supporting the selection of candidate genes mapping in quantitative loci or resulting from association studies. Fine mapping strategies and functional approaches are then required to assess whether the selected genes play a role in determining the analyzed traits.

In the present study, we took advantage from the annotation of the Svevo genome to retrieve all genes mapping under the identified QTLs. As a first step we considered genes already functionally characterized as yield-related in crop species. The attention was on 38 genes distributed in 15 QTL groups. Furthermore, the absence of already characterized yield genes located in the QTL group13_chr5B (NSPK and SPL) drove us to consider the complete list of genes annotated for that region. These genes were further characterized by comparative gene sequence analysis and expression data of the bread wheat homologous genes.

As expected, in the QTL groups representing the variation in flowering time (groups 1, 4 and 17), we found genes already described as contributing directly or indirectly to heading. This is the case of *TRITD2Av1G019250* (*Ppd-A1*) in group 1, *Ppd-B1* in group 4, and *TRITD7Bv1G032180* (*SVP-B2*), *TRITD7Bv1G049470* (*CONSTANS-B1*) and *TRITD7Bv1G129650* (*Ehd3-B*) in group 17. Considering these genes, missense variants have been identified in the coding region of *Ppd*-A1, *SVP-B2* and *CONSTANS-B1* (**Supplementary Table 7**). No QTL for spike architecture is included in group1_chr2A and group4_chr2B, suggesting that in our mapping population and field conditions phenology variability related to main regulatory genes of wheat phenology (*Ppd-A1* and *Ppd-B1*) did not have significant effect on spike/spikelet fertility traits. However, some QTL groups related to floret and spikelet number span regions where genes with possible role in phenology have been annotated (*PHYB, PHYC, CONSTANS-B2, Ta-FDL-6B, VRN-A1, SVP-A1, SVP-A3*). This is the case of the *PHYB* (*TRITD4Av1G061390*) and *PHYC* (*TRITD5Av1G204500*) genes which are known as flowering promoting factors in long day conditions (Kippes et al., 2020) and mapped in QTL groups controlling spike length and number of florets, respectively. An analogous observation supports the *CONSTANS*-B2 gene (*TRITD6Bv1G170830*) that modulates the photoperiod response in wheat, by interacting with *PHOTOPERIOD1* and *CONSTANS1*, and that co-located with QTL group15_chr6B (SPL, SPW, NSPK). In group12_chr5B (SPL, NSPK and SPK), the flowering related factor *TaFDL-6B* was found, which was described as a regulator of the *VRN1* gene (Li and Dubcovsky, 2008). The comparison of the genome sequence of Svevo and MG5323 identified nucleotide variants in the ATG upstream region of *PHYC*, in the gene and putative regulating region of *CONSTANS-B2* and a missense variant with an expected moderate effect were identified for the *SVP-A1* gene.

Other genes influencing both heading time and inflorescence development and mapping under our QTL groups are *VRN-A1* (*TRITD5Av1G204680*) in group10_chr5A (NFRT, FRT) and members of the SVP gene family as *SVP-A1* (*TRITD6Av1G192540*) and *SVP-A3* (*TRITD4Av1G000900*) in groups related to floret number (groups 6 and 14). Focusing on the coding sequence of these genes,; more in-depth analysis is necessary to correlate these variants with the phenotypic variation at the target traits and to verify whether these genes might contribute to the determination of specific yield components in our RIL population.

In the QTL groups specifically controlling floret number (group 2, 6, 8, 10 and 14) we identified 12 functionally characterized genes. Focusing the attention on the coding sequence, missense variants with an expected moderate effect have been identified for the transcription factor coding gene *HOX2* (*TRITD2Av1G066050*) and the cell cycle regulator *KRP1A* (*TRITD6Av1G195200*), located in groups 2 and 14, respectively. In group2_chr2A, an interesting SNP was identified in the coding sequence of the gene *TaCWI-A1* (*TRITD2Av1G179620*) which codes for a cell wall invertase factor. The activity of the protein might be impaired in MG5323 due to the predicted loss of the start codon; in literature, other *TaCWI* genes, as those mapping on chromosomes 4A, 5B and 5D, were reported to influence the TKW (Jiang et al., 2015).

Most of the known genes mapping under the NFRT/FRT QTL groups code for transcription factors belonging to the MADS-box gene family (*FUL-A3, FUL-A2, SVP-A3, VRN-A1, SEP1-6A/PAP2*); other transcription factors belong to the SPL (*SPL13-2A*) gene family, beside the homeobox (HOX2) gene family mentioned above. The MADS-box factors *FUL-A3, FUL-A2*, and *VRN-A1* have been recently reported as crucial factors controlling the identity of the inflorescence meristem; later on, these genes are required for spikelet differentiation and for the spike determinate growth (Li et al., 2019). A further MADS-box coding gene mapping in the group10_chr5A, identified for floret number, is the *SEP1-6A* gene (*TRITD5Av1G204770*). This gene is reported as the homologous of the rice gene *OsMADS34* controlling spikelet number and morphology (Lin et al., 2014; Schilling et al., 2020). Nucleotide differences have been identified when comparing the Svevo and MG5323 genomes for most of the mentioned genes, with most of the SNPs mapping outside the coding region (**Supplementary Table S7**). Notably, polymorphisms in the promoter region could be responsible for differential gene expression that is quite relevant for the activity of transcription factors.

In our analysis, loci influencing spike-related traits are distributed in 7 QTL groups where we have identified 25 genes already characterized as yield-related genes (**Supplementary Table S7**); those mainly involved in spike development are *LEAFY-like* (*TRITD2Av1G254900*), *CEN4-A* and *CEN5-B* (*TRITD4Av1G228450*, *TRITD5Bv1G078820*), *Q* (*TRITD5Av1G231510*), *KRP1-B* and *KRP-B2* (*TRITD6Bv1G188320* and *TRITD7Bv1G030760*) and *WAPO-A1* (*TRITD7Av1G253750*) genes. Focusing on this subset of genes, we have detected nucleotide polymorphisms in the gene sequence of *CEN4-A, KRP1-B, KRPB2* and *WAPO-A1*. *CEN* genes are also considered florigen antagonist genes and the ectopic expression of *CEN2* was recently associated with repression of the spikelet meristem identity; in the parental lines used for this work, we identified 5 SNPs in the upstream region of the *CEN4-A* gene which might influence the expression profile of the gene. The *KRP1-B* and *KRPB2* genes play a role in cell cycle control and their effect on influencing the weight, size, and number of organs is demonstrated by the fact that plants carrying mutations in these genes have been developed and are protected by US patents (US 2016/0002656A1). Nucleotide polymorphisms have been identified in both the genes comparing Svevo and MG5323 genomes. Another relevant candidate gene mapping under a QTL group involved in SPK and NSPK is the *WAPO-A1* gene, which encodes a F-box protein. Recently, the *WAPO-A1* gene was described as a causative gene of spikelet number per spike in wheat. The SNP identified in the coding region of the *WAPO-A1* gene was already described in literature but based on the BLOSUM 62 score it is not predicted to affect the protein structure or function (Kuzay et al., 2022).

Interestingly, our analysis led to the identification of a novel region influencing the number of spikelets and spike length on chromosome 5B (QTL group13). Considering the genes mapping in this QTL group, we created a sub-list of the most interesting genes using the expression profile of the bread wheat homologous genes as a selection criterium (**Supplementary Table S10**) and here the most interesting genes are discussed Combining the expression data with the SNPs analysis led us to disregard the spike-specific genes as all of them share identical sequences in the parental lines. Considering the spike and grain specific genes, most of the SNPs are in the promoter regions except for the *TRITD5Bv1G184470* gene, coding for a putative transport protein carrying 11 transmembrane helices, which has two missense variants in the coding sequence. The only spike-enriched gene codes for a cytokinin riboside 5’-monophosphate phosphoribohydrolase, belonging to the *LOG* gene family; interestingly, *LOG* genes in rice and wheat have been associated to the regulation of meristem maintenance thus influencing yield components (Chen et al., 2022). Considering the genes that are expected to be repressed in the spike, we selected 4 genes with only the *TRITD5Bv1G182420* showing a SNP in the upstream region. This gene codes for a BURP domain-containing protein; this is a plant-specific class of protein involved in many aspects of plant differentiation; in some cases, an association with cell wall properties and cell elongation mechanisms was reported (Ding et al., 2009; Park et al., 2015).

Finally, the comparison with Valladares García et al. (2023), where the same segregant population was used, allowed us to evaluate the two main yield components (namely, grain number and grain weight). We highlighted two regions overcoming the trade-off between TKW/grain size and grain number: two genes can be proposed as putatively playing a central role: *CENTRORADIALIS 4* (*CEN4-A*) and *BIG GRAIN PROTEIN 1* (*BG1*) for 4A and 4B regions, respectively (Valladares García et al., 2023). The genome sequence comparison between MG5323 and the durum wheat reference genome highlighted only polymorphisms in the upstream gene sequences for both genes (4 for *CEN4-A* and 1 for *BG1*) and a future detailed analysis will be required to understand their possible role. Importantly, it may be concluded that these haplotypes and their functional markers could be utilized in marker-assisted selection for breeding high-yielding varieties.

## Conclusion

The current study contributes to lay the foundations on understanding the genetic bases of spike fertility, looking at spikelet number per spike and floret number per spikelet, by identifying different genomic regions involved in their definition for which stable QTLs for both traits have been detected. A fine mapping approach will be required to determine the candidate gene associated to the spikelet number per spikes (QTL group13_chr5B), where known genes are not well characterized for their role in spike architecture. Finally, the haplotypes of the two regions where the trade-off between TKW/grain size and grain number are overcome could be considered in marker-assisted selection for breeding high-yielding varieties.

This work can play an important role in understanding the evolutionary dynamics occurred during the domestication among emmer and durum wheat genomes, assisting the efficiency of plant breeding programs and facilitating the development of new cultivars with increased grain yield.

## Data Availability

The datasets presented in this study can be found in online repositories. The names of the repository/repositories and accession number(s) can be found below: https://www.ebi.ac.uk/ena, PRJEB63365.
