# Supplementary material for: Independent genetic factors control floret number and spikelet number in Triticum turgidum ssp
Source: Front Plant Sci. 2024 Aug 26;15:1390401. doi: 10.3389/fpls.2024.1390401 (PMC11381284; doi:10.3389/fpls.2024.1390401)
Supplement: Supplementary file 1 [file DataSheet1.docx]

**Supplementary Figures**

**Supplementary Figure 1**. Pearson’s correlation among traits recorded from dry (_d) and fresh (_f) spikes. Correlation plot for F19 (A), F20 (B), F21 (C), P20(D) and for multi-environment (E) are reported. The traits are denoted as: FRT, total floret number; NFRT, net floret number; SPK, total spikelet number; NSPK, net spikelet number.


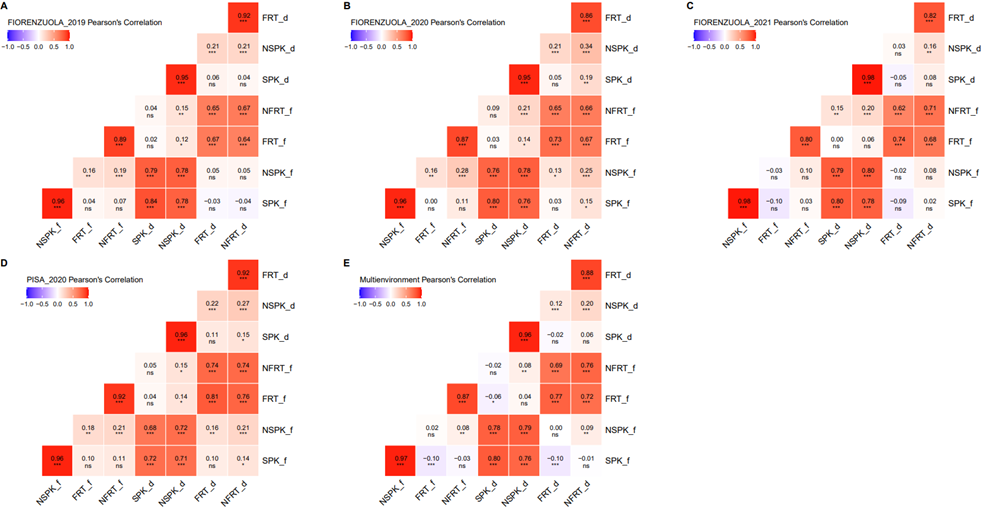


* Significance level at *p*<0.05, **significance level at *p* <0.01, ***significance level at *p* <0.001 and ns for non-significant.

**Supplementary Figure 2.** Pearson’s correlation among all traits in each environment. Correlation plot for F19 (A), F20 (B), P20 (C) and F21(D) are reported.


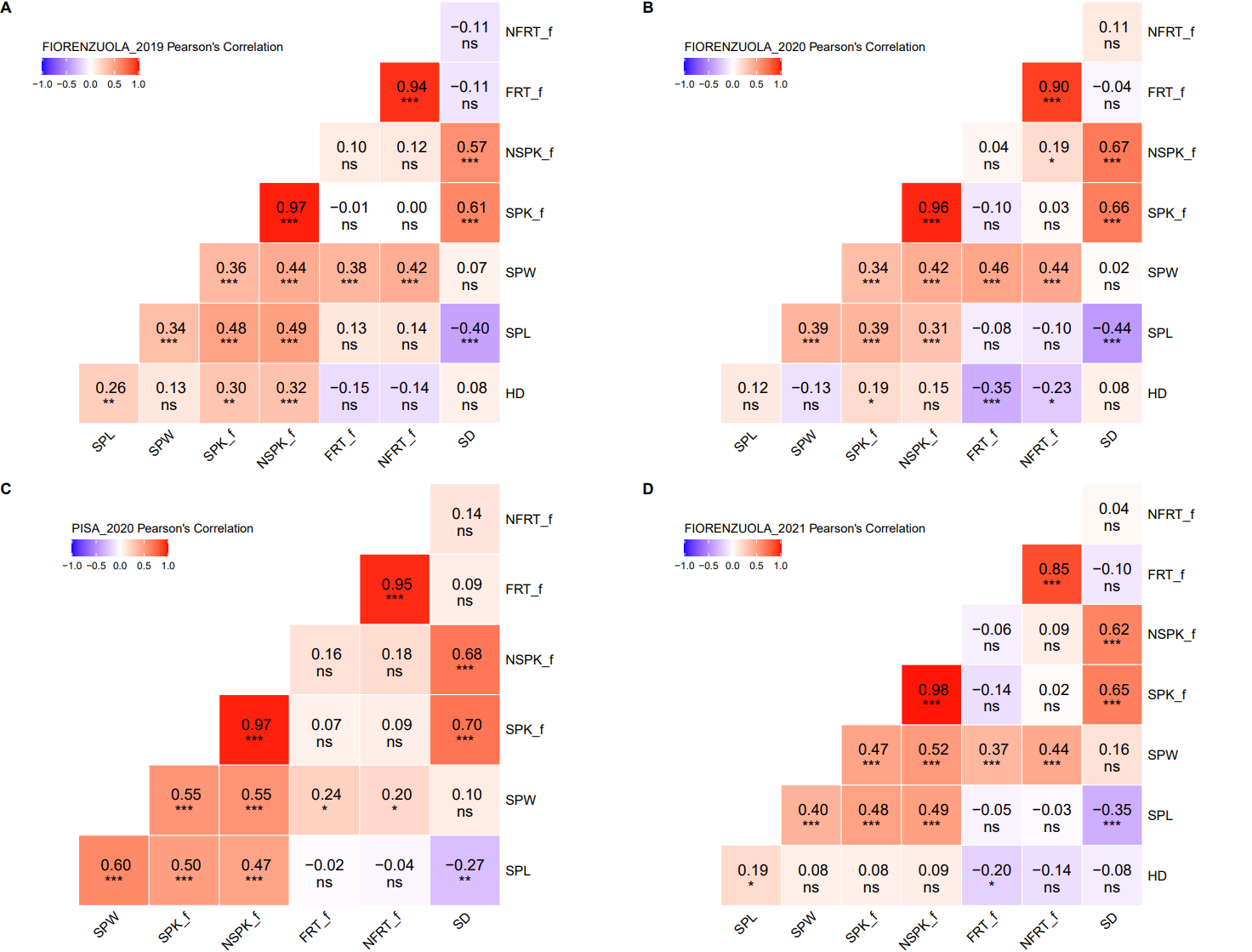


* Significance level at *p* <0.05, **significance level at *p* <0.01, ***significance level at *p*<0.001 and ns for non-significant.

The traits are denoted as: FRT, total floret number; NFRT, net floret number; SPK, total spikelet number; NSPK, net spikelet number.

**Supplementary Figure 3.** A) Principal component analysis of multi-environment BLUP for all traits; dots are coloured according heading date. B) Correlation of the principal components with all targeted traits.


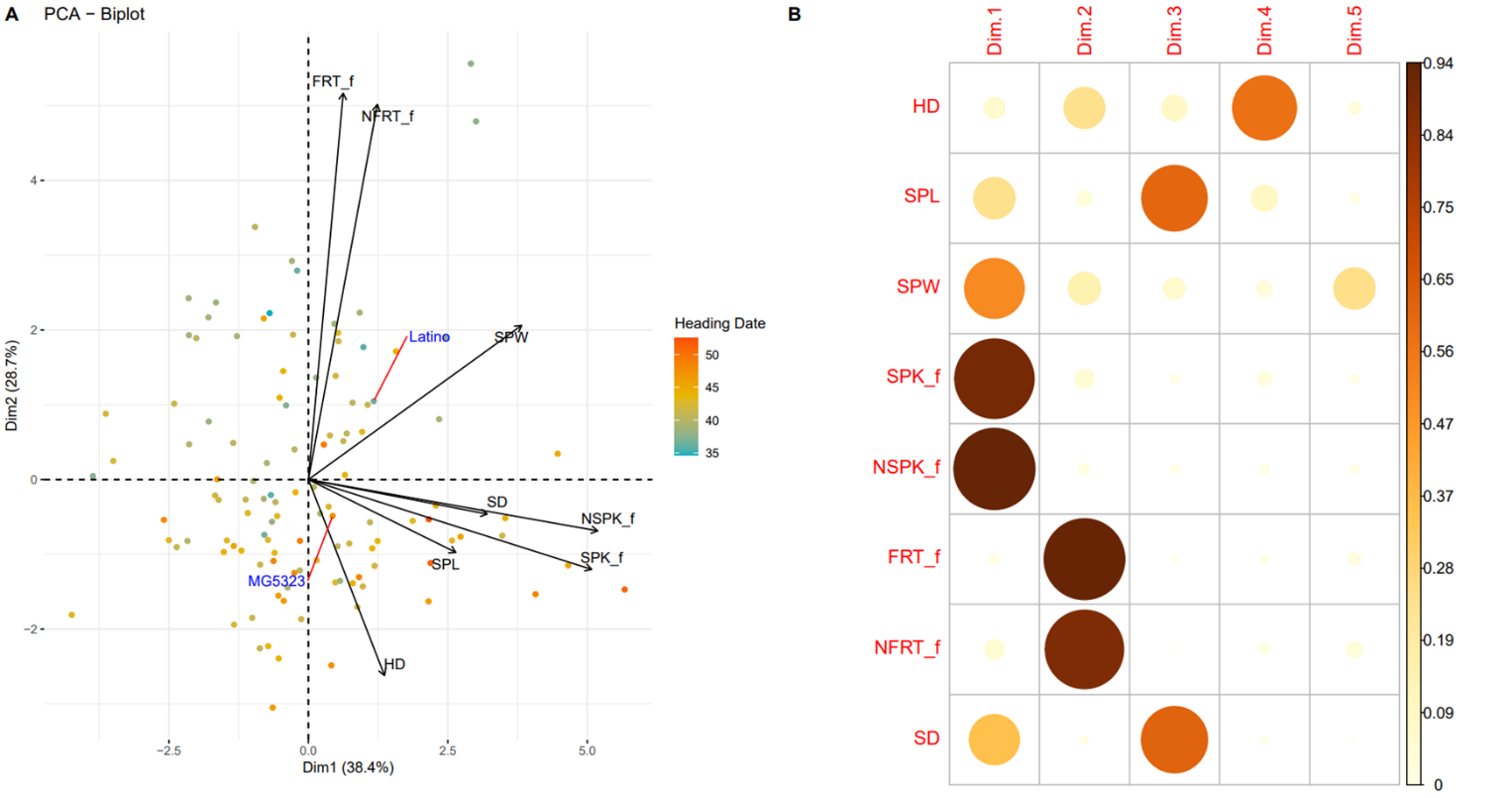


The traits are denoted as: FRT, total floret number; NFRT, net floret number; SPK, total spikelet number; NSPK, net spikelet number.
